# Antigenic assessment for the β2-glycoprotein I/Platelet factor 4 complex in thrombotic patients with antiphospholipid syndrome

**DOI:** 10.3389/fimmu.2025.1674181

**Published:** 2026-01-12

**Authors:** Francesca Villani, Antonella Capozzi, Federica Maria Ucci, Simona Truglia, Manuela De Michele, Silvia Mancuso, Luca Rapino, Giuseppe Tripodi, Valeria Manganelli, Gloria Riitano, Roberta Misasi, Maurizio Sorice, Antonio Sili Scavalli, Fabrizio Conti, Cristiano Alessandri

**Affiliations:** 1 Department of Translational and Precision Medicine, “Sapienza” University of Rome, Rome, Italy; 2 Department of Experimental Medicine, “Sapienza” University of Rome, Rome, Italy; 3 Rheumatology Unit, Department of Clinical Internal, Anesthesiological and Cardiovascular Sciences, “Sapienza” University of Rome, Rome, Italy; 4 Emergency Department, “Sapienza” University of Rome, Rome, Italy; 5 Wellbeing, Health and Environmental Sustainability Department “Sapienza” University of Rome, Rieti, Italy

**Keywords:** antiphospholipid syndrome, COVID-19, platelet factor 4, thrombosis, vaccine-induced immune thrombotic thrombocytopenia (VITT), β2-glycoprotein I

## Abstract

**Objective:**

Antiphospholipid syndrome (APS) is an autoimmune condition characterized by recurrent thrombosis, pregnancy-related complications and circulating antiphospholipid antibodies, including anti-β2-glycoprotein I (β2-GPI). Platelet factor 4 (PF4) is a pro-coagulant protein expressed by activated platelets. It is considered a potential platelet ligand for oxidized β2-GPI, and this interaction may play a role in the thrombotic manifestations of APS. This study aims to assess β2-GPI/PF4 complex autoantibodies in sera of thrombotic patients with APS and their potential functional role in platelet activation.

**Methods:**

We analysed sera from 73 patients with thrombotic APS, 20 with obstetric APS, 20 with systemic lupus erythematosus (SLE), 20 with non-APS thrombosis, 3 with vaccine-induced immune thrombotic thrombocytopenia (VITT), 20 with COVID-19 and 45 healthy donors (HDs). These samples were tested by ELISA for antibodies to the β2-GPI/PF4 complex after *in vitro* induction of spontaneous β2-GPI protein oxidation.

**Results:**

Anti-β2-GPI/PF4 were detected in 34.24% of thrombotic APS patients and 20% of obstetric APS. All VITT and none of the SLE, non-APS thrombosis, COVID-19 patients and HDs were positive for anti-β2-GPI/PF4. In thrombotic APS, a significant association was found between anti-β2-GPI/PF4 positivity and antibody titer with venous thrombotic complications (p = 0.032, p = 0.01) as well as between anti-β2-GPI/PF4 and triple positivity to conventional aPLs (p = 0.028). Notably, antibody titer correlated with the young age both at diagnosis (p<0.001) and at the current evaluation (p=0.001). Moreover, HDs’ platelets, *in vitro* treated with Ig fractions from APS patients, exhibited a significant increase in phospho-ERK and phospho-p38 expression, leading to NF-κB activation and TF expression.

**Conclusion:**

This study demonstrated the presence of anti-β2-GPI/PF4 in patients with APS, where it may be involved in the mechanism underlying the hypercoagulable state and correlated with a greater risk of developing thrombosis, especially in the young population.

## Introduction

Antiphospholipid antibody syndrome (APS) is an autoimmune, multisystemic disorder characterized by vascular thrombosis and pregnancy morbidity associated with the presence of antiphospholipid antibodies (aPLs) (1–4). The pathophysiology of the disease is based on the action of autoantibodies, which engage phospholipids and phospholipid-binding proteins at cell surfaces, thereby activating endothelium, platelets, and leukocytes, which leads to the formation of *in situ* thrombosis and promotes other autoimmune and inflammatory complications (5, 6). aPLs, which include anti-β2-glycoprotein I (β2-GPI), anti-cardiolipin antibodies (aCL) and Lupus anticoagulant (LAC), are serological markers of APS (7). β2-GPI, also known as apolipoprotein H, is the main antigenic target of aPLs (8). Several clinical, epidemiological and *in vivo* studies reported anti-β2-GPI antibodies as a significant risk factor for thrombotic morbidity. β2-GPI is a 48-kDa plasma protein composed of 326 amino acid residues deployed in five domains (9–11). Domain I interacts with domain V in plasma, forming a circular structure, but the binding of the positively charged lysine cluster on β2-GPI domain V to negatively charged phospholipids extends the molecule into a fishhook configuration, exposing cryptic epitopes in domain I. In this open conformation, β2-GPI becomes available for binding to the pathogenic autoantibodies (12). β2-GPI is a physiological component of human blood, and it is involved in several biological pathways, including coagulation and inflammation. However, it can become a target of pathogenic autoantibodies; its immunogenicity is mainly ascribed to the oxidation of the terminal sulfhydryl groups, leading to the formation of oxidized β2-GPI (oxβ2-GPI), and other post-translational modifications, such as glycation, acetylation or carbamylation (13–15).

In this regard, a previous study has demonstrated a role for platelet factor 4 (PF4) in stabilizing the dimeric form of β2-GPI and subsequent binding to anti-β2-GPI antibodies, as well as to exposed phospholipids and receptors on the platelet surface. A role for PF4 in the stabilization of the dimeric form of β2-GPI and subsequent binding to anti-β2-GPI antibodies on the platelet surface has been demonstrated (16). PF4 is a member of the C-X-C chemokine family secreted by α-granules of platelets during their activation and aggregation, in a protein kinase C-dependent manner. It can promote blood coagulation by modulating the anti-thrombotic effects of heparin-like molecules. PF4 can bind to the platelet surface and has a high affinity for heparin and other anionic glycosaminoglycans (GAGs) on endothelial cells or platelet surfaces (17–19). Therefore, it has a proven procoagulant role in inhibiting heparin-dependent acceleration of thrombin inactivation by antithrombin (20) and promoting platelet aggregation (21). Moreover, a tetramer of PF4 selectively binds two molecules of β2-GPI, favoring their dimerization, which is crucial in platelet activation and therefore in thrombotic manifestations of APS. It has been demonstrated that the positively charged surface of the PF4 tetramer interacts with the negatively charged regions of β2-GPI domains, stabilizing the link between the antigen and the phospholipid surface, thereby increasing the likelihood of binding with the respective antibodies (16). In this concern, Sikara et al. demonstrated by silico molecular docking, that β2-GPI forms stable complexes with PF4, and then, using size exclusion chromatography, they highlighted that anti-β2-GPI antibodies selectively interact with complexes composed of β2-GPI dimeric structure and PF4 tetramer (16). These complexes can become thrombogenic, activating platelets, as further confirmed by data of p38MAP kinase phosphorylation and thromboxane B2 production (16). Some findings reported, by electrophoretic analysis, that the dimeric form of β2-GPI was present in variable amounts of the oxidized protein, but it was absent in the native one (22). Thus, β2-GPI may interact, after dimerization, because of oxidation conformational changes, and the dimeric form of the protein would represent oxβ2-GPI (23, 24).

PF4 is a key antigen in heparin-induced thrombocytopenia (HIT), which shares similar clinical manifestations with APS, such as thrombocytopenia and thrombosis. HIT can have severe life‐ and limb‐threatening complications and is characterized by strong platelet activation (25). However, PF4 is not immunogenic in its primary form but needs conformational changes to expose a neo-epitope, forming the HIT antigen. These changes occur by the formation of complexes between PF4 and negatively charged molecules, of which heparin represents the most frequent, but not the only one (26). PF4/heparin complex formation results in a highly immunogenic response, inducing anti-PF4/heparin antibodies in a minority of heparin-treated patients. In the presence of these autoantibodies, platelets were mainly activated through FcγRIIa receptors [immunoglobulin G (IgG) receptors (FcγRIIa)], which are their unique receptor for IgG antibodies (27). Therefore, PF4 may be a common antigenic link in syndromes such as APS, HIT and vaccine-induced immune thrombotic thrombocytopenia (VITT). Interestingly, in several patients, unusual thrombosis and thrombocytopenia may be associated with the presence of anti-PF4 antibodies, even in the absence of prior heparin therapy. It could only be explained by HIT antibodies with heparin-independent platelet-activating properties (28, 29). Recent studies have shown structural similarities between heparin and β2-GPI, which may be responsible for thrombotic events in those patients who had never taken heparin (30). More specifically, considering these structural similarities, β2-GPI may mimic heparin and bind to PF4. The evidence demonstrating the immunogenicity of oxβ2-GPI/PF4 could explain the thrombotic events following vaccination in subjects who have never received heparin, as well as in APS.

In light of these premises, we aimed to test β2-GPI/PF4 complex autoantibodies in sera of thrombotic patients with APS and their potential functional role in platelet activation. Identifying these antibodies may offer new diagnostic or prognostic insights, particularly in stratifying APS patients with a higher risk of early and recurrent thrombotic events.

## Materials and methods

### Patients

The study included 73 consecutive thrombotic APS patients classified according to Sidney criteria (7) and referred to the Lupus Clinic, Rheumatology Unit of Sapienza University of Rome from 2020 to 2022. Demographic, clinical and laboratory patient features were collected and registered in electronic medical records.

As control groups, patients with obstetric APS, systemic lupus erythematosus (SLE), non-APS thrombosis, COVID-19 with or without vascular thrombosis and healthy donors (HDs), matched for sex and age, were included. We also enrolled patients with VITT, referred to the Emergency Department of Umberto I Polyclinic in Rome, who had malignant middle cerebral artery (MCA) infarction and concomitant thrombocytopenia within 10 days after vaccination with ChAdOx1 nCoV-19.

At enrollment, we recorded cardiovascular risk factors (hypercholesterolemia, smoking, hypertension, diabetes) and extra-criteria manifestations from patients and controls. Sera were collected and stored at - 20 °C. Plasma samples were used for the LAC test.

The study was conducted in accordance with the Declaration of Helsinki and was approved by the local Ethics Committee at Sapienza University of Rome (No. 0586-20). All the patients and HDs gave written informed consent.

### Detection of aPLs

Serum aCL and anti-β2-GPI (IgG/IgM) antibodies were detected employing a commercial Enzyme-linked immunosorbent assay by the QUANTA Lite detection kit using QUANTA-Lyser 3000 system (Inova Diagnostic Inc., San Diego, California, USA), according to the manufacturer’s instructions. All the obtained results were also confirmed by a chemiluminescence assay using the QUANTA Flash detection kit on a BIO-FLASH system (Inova Diagnostic Inc.). In addition, LAC was evaluated by two coagulation systems involving the use of a dilute sensitized activated partial thromboplastin time (aPTT) and a dilute Russell’s viper venom time (dRVVT), followed by a confirmation test (Hemoliance Instrumentation Laboratory, Lexington, MA, USA).

### Detection of antibodies directed to β2-GPI/PF4 complex by ELISA

The antigenicity of β2-GPI/PF4 complex was tested with a modified anti-β2-GPI ELISA according to Sikara et al. (16). Briefly, recombinant β2-GPI (10 μg/mL diluted in 0.05 M NaHCO_3_ buffer, pH 9.5) (Calbiochem, La Jolla, CA, USA) was used in the amount of 50 μL/well. To coat polystyrene microtiter plates, β2-GPI was used in the absence of protective reducing agents and kept at room temperature to induce “*in vitro*” spontaneous oxidation by oxygen in the air, as previously reported (31, 32). Thus, electrophoresis and Western blot analysis of β2-GPI preparations showed that native β2-GPI and spontaneously oxidized β2-GPI produced different protein profiles, as previously described (32). After coating and incubation overnight at 4 °C, the plates were washed 5 times with phosphate buffer saline (PBS)/0.1% Tween 20 (PBS-T) and blocked for 2 hours with 2% Bovine Serum Albumin (BSA)/PBS-T, at room temperature (RT). Then, the wells were washed again as described before and incubated overnight at 4 °C with 50 μL/well of PF4 (2.0 μg/mL in blocking buffer) (R&D Systems, Minneapolis, MN, USA). After 5 washes as above, the wells were incubated with sera from patients (dilution 1:100, in blocking buffer) for 2 hours at RT. Afterwards, the plates were washed and incubated for 1 hour at RT with 100 μL/well of horseradish peroxidase-conjugated anti-human IgG antibodies (Sigma-Aldrich, Milan, Italy), diluted in the blocking buffer. The plates were washed as above, and the bound peroxidase was then revealed by adding 100 μL/well of O-phenylenediamine dihydrochloride buffer and stopping color development with H_2_SO_4_ 0.2 M for 5 min. The absorbance of the wells was measured with a microplate reader at 492 nm. In parallel, all sera were analyzed with the same procedure but without coating with β2-GPI/PF4 complex. Intra- and interassay variations were determined by assaying two APS samples 16 times in one assay (intra-assay) and assaying the two samples in quadruplicate in four consecutive assays (interassay). Data were analyzed as the mean optical density (OD) corrected for background (wells without coated antigens). Cut-off values were calculated using the mean of optical density ± 2 S.D. of 45 HDs.

### Detection of anti-PF4 antibodies

All the patients’ and HD’s sera were tested for antibodies against PF4. Samples were analyzed using a commercial enzyme immunoassay (Immucor, Lifecodes, Waukesha, WI), according to the manufacturer’s instructions. Results were acquired and analyzed based on an OD threshold measurement of 0.40 following manufacturer guidelines and after validation with internal positive and negative controls.

### Absorption test

Serum of VITT patients and HDs were subjected to an absorption test. Sera from VITT patients, positive for both anti-PF4 and anti-β2-GPI/PF4 complex antibodies, were diluted 1:100 in the blocking buffer (2% BSA/PBS-T) and incubated with PF4 (100 μg/mL) for 1 h at 37°C and then overnight at 4°C. The mixture was centrifuged for 15 min at 27, 000 x g at 4°C. The supernatant fractions were collected as absorbed sera and then analyzed for the presence of anti-β2-GPI/PF4 or anti-PF4 antibodies by ELISA, as reported above. Absorbances of unabsorbed patient sera were set to 100%. The results were expressed as a percentage of reactivity obtained from the ratio between the absorbance values of the absorbed sera and those of the unabsorbed sera.

### Platelet preparation and treatment

Blood samples of HDs, containing acid citrate dextrose (ACD) as an anticoagulant, were used to prepare platelets. HDs signed the informed consent from the Transfusion Center of the Policlinico Umberto I, Sapienza University of Rome.

Platelet rich plasma (PRP) was initially separated from the whole blood by centrifugation at 150 × g for 15 min at 20°C. Two-thirds of the PRP was transferred to a new sterile tube, with ACD added to prevent platelet activation and maintain the integrity of the buffy coat layer, thereby avoiding contamination. After PRP centrifugation, at 900 x g for 10 min at 20°C (with no brake applied), platelet-poor plasma (PPP) was discarded, and the obtained platelet pellets were resuspended in Tyrode’s buffer, containing 10% (v:v) ACD. Subsequently, after washing, as above, platelet pellets were resuspended in Tyrode’s buffer, in the presence of BSA 3 mg/ml.

Platelets were counted by a hemocytometer (Coulter, Beckman Coulter, Brea, California, USA), which showed that leukocyte contamination was < 1 leukocyte/10^7^ platelets. The purity of the isolated platelets was verified and confirmed by flow cytometric analysis (CytoFLEX, Beckman Coulter) after fluorescein isothiocyanate (FITC)–conjugated anti-CD41 antibody (Beckman Coulter) staining (data not shown).

IgG fractions were isolated from the 5 sera of APS patients, selectively positive for anti-β2-GPI/PF4, but negative for anti-β2-GPI antibodies, using (NH4)_2_SO_4_ (ammonium sulfate, Sigma-Aldrich) precipitation, slightly modified. A preliminary purity reduction step was performed by precipitation with caprylic acid (octanoic acid, Sigma-Aldrich), followed by the addition of saturated (NH4)_2_SO_4_ to a final concentration of 33%, which was then slowly added to the sera and incubated for 1 h at 4°C. The samples were centrifuged at 3000 x g for 30 min at 4°C, and the supernatant was carefully transferred into a fresh tube. Subsequently, a saturated solution of (NH4)_2_SO_4_ was added to bring its final concentration to ∼approximately 50% and incubated for 1 h at 4°C. The IgG were finally obtained by centrifugation at 3000 x g for 30 min at 4°C after removing the supernatant. The pellets were resuspended with PBS equal to the original volume of serum. As a final step, samples were dialyzed overnight against (NH4)_2_CO_3_ (ammonium carbonate) to remove remaining ammonium sulfate and then lyophilized and resuspended in sterile PBS. As a control, we used IgG from human normal serum (Sigma-Aldrich).

After isolation, platelets (3 × 10^8^/mL), were seeded in into 6-well cell culture plates and incubated with IgG fractions (200 μg/mL) from sera of APS patients or with IgG fractions from human normal serum for 10 min to analyze phospho-ERK, phospho-p38 and phospho-p65 activation or for 4 hours to evaluate Tissue factor (TF) expression.

### Western blot analysis

After the treatment, platelets were lysed using a buffer prepared with 20 mM HEPES, pH 7.2, 1% Nonidet P-40, 10% glycerol, 50 mM NaF, 1 mM Na_3_VO_4,_ and a protease inhibitor cocktail (Sigma-Aldrich). Equal amounts of protein extracts were analyzed by Western blot. Initially, equal amounts of lysate samples were subjected to 10% sodium dodecyl sulfate polyacrylamide gel electrophoresis (SDS-PAGE). Then they transferred to polyvinylidene difluoride (PVDF) membranes (Bio-Rad Laboratories, Richmond, CA, USA). Membranes, blocked with Tris-buffered saline Tween 20 (TBS-T) 3% BSA, were incubated with primary polyclonal antibodies: rabbit anti-phospho-ERK1/2, rabbit anti-phospho-p38, rabbit anti-phospho-NF-κB-p65 antibodies (Cell Signaling, Inc., Danvers, MA, USA) or rabbit anti-TF (Abcam, Cambridge, UK). Then, membranes were incubated with horseradish peroxidase (HRP)-conjugated anti-rabbit IgG (Sigma-Aldrich) as secondary antibodies. Finally, to visualize antibody reactions, an enhanced chemiluminescence Western blot system (Amersham Pharmacia Biotech, Buckinghamshire, UK) was used. To adjust for total protein content, phospho-ERK1/2, phospho-p38, phospho-NF-κB-p65 and TF membranes were stripped and reprobed with rabbit anti-ERK1/2, rabbit anti-p38, rabbit anti-NF-κB-p65 (Cell Signaling, Inc.), mouse anti-β-tubulin or mouse anti-β-actin (Sigma-Aldrich). Densitometric scanning analysis was performed using a NIH Image 1.62 software (National Institutes of Health). The density of each band (absolute value) in the same gel was analyzed.

### Statistical analysis

Data are expressed as mean (S.D.) or median (interquartile range) according to the distribution of values. Categorical data were represented as frequencies and proportions. Chi-square test or Fisher’s exact test was used to compare categorical variables. Mann-Whitney U test was used for between-group comparisons of non-normally distributed continuous data, and Spearman’s test was used for correlations between non-normally distributed variables. For western blot, the Shapiro–Wilk test was applied to test data normality. Normally distributed data were analyzed using an exploratory one-way-ANOVA; *post-hoc* tests with appropriate multiple comparison correction were used if the ANOVA was significant. A p value less than 0.05 was considered as significant. Statistical analysis was performed using the Prism version 7 (GraphPad Software, San Diego, California, USA). ROC curve analysis was additionally used to explore the discriminative capacity of anti-β2-GPI/PF4 levels in identifying thrombotic APS patients.

## Results

### Detection of anti-β2-GPI/PF4 antibodies

We enrolled 73 thrombotic APS [mean age 53.6 (S.D. 13.6) years; 22 male, 51 female]. As controls, we analysed 20 obstetric APS patients [mean age 36.5 (S.D. 7.7) years], 3 VITT patients [mean age 57 (S.D. 2) years; 1 male, 2 female], 20 SLE patients [mean age 42 (S.D. 12.5) years; 2 male, 18 female], 20 non-APS thrombotic patients [mean age 41.1 (S.D. 11.76) years; 6 male, 14 female], 10 COVID-19 patients with thrombosis [mean age 72.8 (S.D. 17.6) years; 7 male, 3 female], 10 COVID-19 patients without thrombosis [mean age 68.5 (S.D. 15.8) years; 7 male, 3 female] and 45 HDs [mean age 41.5 (S.D. 12.1) years; 7 male, 38 female]. All clinical, demographic and laboratory characteristics of patients are reported in **Table 1** (APS) and **Supplementary Table 1** (VITT). At the time of the first event, none of the patients were taking anticoagulant therapy; patients were only taking warfarin therapy at the time of recurrence.

**Table 1:** Demographics and clinical characteristics of thrombotic APS patients.

Characteristics	Patients n= 73
Female/Male	51/22
Median age in years (IQR)	52 (45.2-61.7)
Clinical features	**n (%)**
PAPS	43 (58.9)
SAPS	28 (38.36)
Catastrophic syndrome	2 (2.74)
Thrombosis	**n (%)**
*Arterial thrombosis*	
Myocardial infarction	11 (15.06)
Ischemic stroke	28 (38.35)
Peripheral thrombosis	42 (57.53)
*Venous thrombosis*	
Deep and superficial venous Thrombosis	41 (56.16)
*Recurrent Thrombosis*	34 (46.57)
Extra-criteria manifestations	**n (%)**
Extra-criteria manifestations	38 (52.05)
Livedo reticularis	10 (13.69)
Thrombocytopenia	13 (17.8)
Migraine	8 (10.95)
Autoimmune haemolytic anaemia	3 (4.1)
Valvulopathy	5 (6.84)
APS nephropathy	3 (4.1)
Conventional cardiovascular risk factors	**n (%)**
Hypercholesterolemia	20 (27.39)
Smoking	22 (30.13)
Hypertension	25 (34.24)
Diabetes	5 (6.84)
Anti-β2-glycoprotein I antibodies IgM +	27 (36.98)
Low title	9 (12.32)
Medium title	10 (13.69)
High title	8 (10.95)
Anti-β2-glycoprotein I antibodies IgG +	43 (58.9)
Low title	7 (9.58)
Medium title	11 (15.06)
High title	25 (34.74)
Anti-Cardiolipin antibodies IgM +	33 (45.20)
Low title	11 (15.06)
Medium title	13 (17.8)
High title	9 (12.32)
Anti-Cardiolipin antibodies IgG +	56 (76.71)
Low title	10 (13.69)
Medium title	20 (27.39)
High title	26 (35.61)
LAC +	33 (45.20)
Triple Positivity	18 (24.65)

APS, (antiphospholipid syndrome); PAPS, (primary antiphospholipid syndrome); SAPS, (secondary antiphospholipid syndrome). The bold value indicate a significant value.

**Figure 1:**
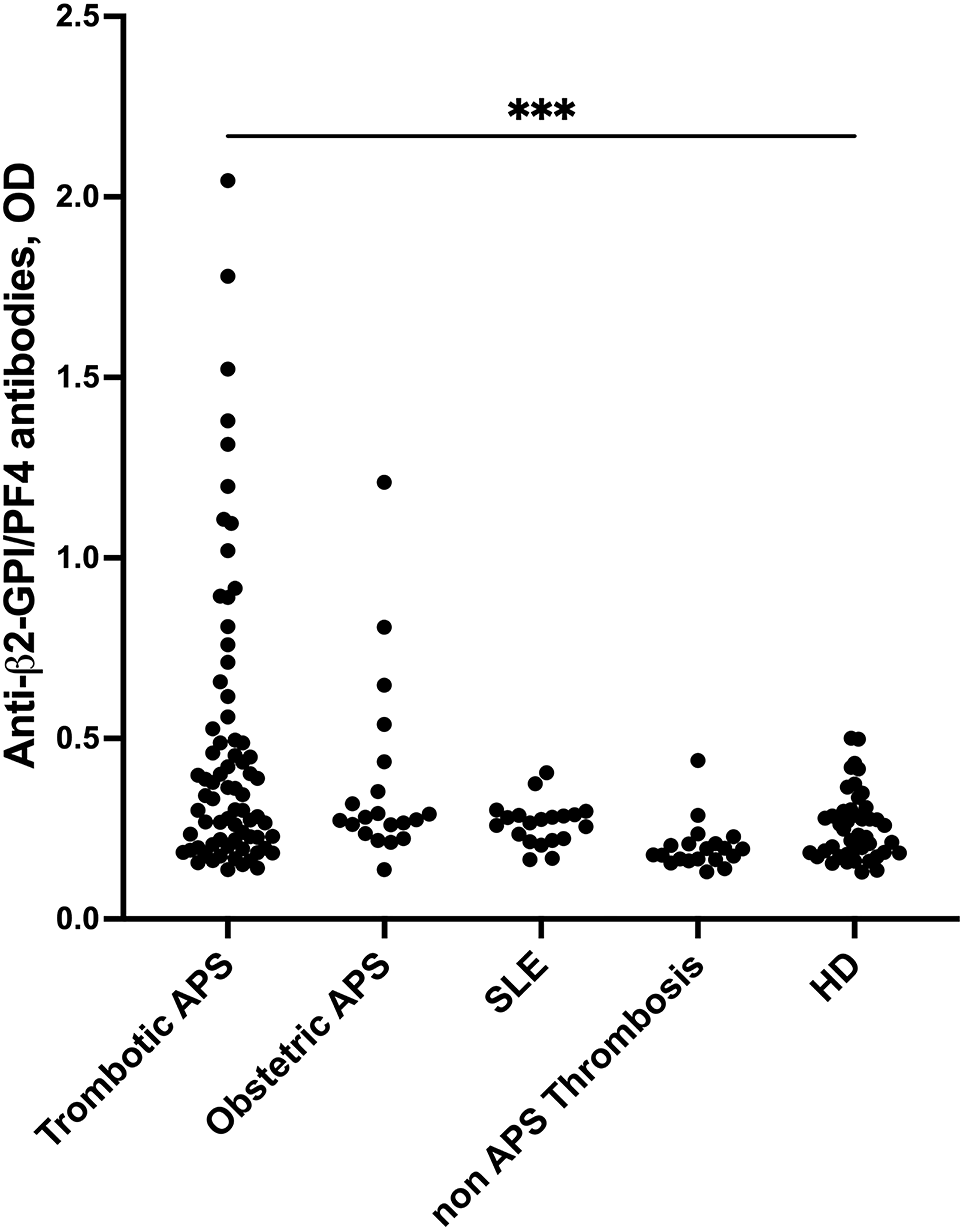
Anti-β2-GPI/PF4 serum levels. Sera from patients with thrombotic APS, obstetric APS, SLE, non-APS thrombosis and HDs were analysed by ELISA for detection of anti-β2-GPI/PF4 antibodies. ***p < 0.001.

In the APS population, 25 out of 73 (34.25%) thrombotic and 4 out of 20 (20%) obstetric patients tested positive for anti-β2-GPI/PF4 antibodies. In comparison, anti-PF4 antibodies were detected only in 3 out of the 73 thrombotic APS patients (4.1%) and in none of the 20 obstetric APS patients (0%), respectively. Overall, the median levels of anti-β2-GPI/PF4 antibodies were significantly higher in patients with thrombotic APS compared to HDs (p < 0.001) (**Figure 1**). None of SLE patients, non-APS thrombotic patients, COVID-19 patients and HDs have been found positive for anti-β2-GPI/PF4 (**Figure 1**). As expected, all three VITT patients were positive for both anti-β2-GPI/PF4 antibodies and anti-PF4 antibodies.

In order to investigate the specificity of the β2-GPI/PF4 assay, we used sera from VITT patients positive for both anti-β2-GPI/PF4 and anti-PF4 antibodies. They were absorbed with PF4 and then analyzed in the anti-β2-GPI/PF4 test. Interestingly, the residual reactivity of the absorbed sera was however appreciable compared to that of the unabsorbed sera; it was on average 65%, demonstrating a specific recognition by anti-β2-GPI/PF4 antibodies (**Supplementary Figure S1**).

**Figure 2:**
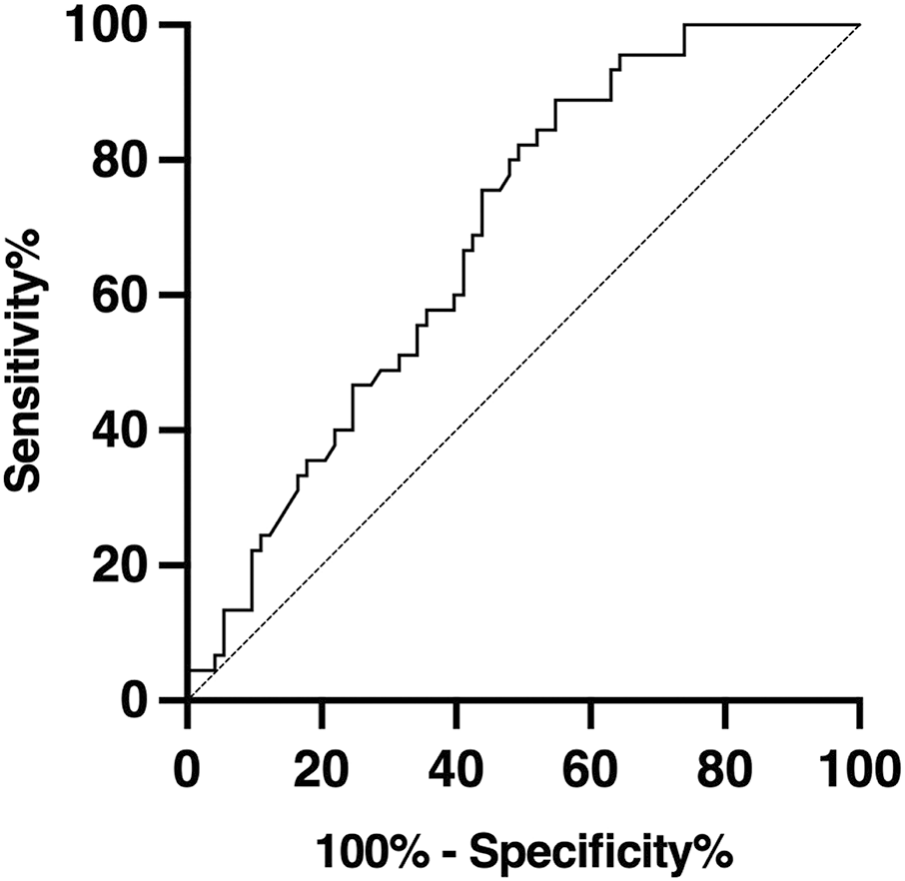
Receiver operating characteristic (ROC) analysis of APS patients. The variation in anti-β2-GPI/PF4 antibody levels presented an area under the curve (AUC) of 0.701.

The receiver operating characteristic (ROC) analysis of the connection between clinical sensitivity and specificity for anti-β2-GPI/PF4 antibodies in APS patients is shown in **Figure 2**. All the results obtained by anti-β2-GPI/PF4 and anti-PF4 antibody tests are shown in **Table 2**. An aPLs heatmap of APS patients, analyzing association of anti-β2-GPI/PF4 with LAC, anti-β2-GPI and aCL, is reported in **Supplementary Figure S2**. A multivariate logistic regression analysis did not find significant correlations.

**Table 2:** Prevalence of autoantibodies in patients and other study groups.

Patients, (n)	Anti-β2GPI/PF4 n (%)	Anti-PF4 n (%)
Thrombotic APS, (73)	25/73 (34.25)	3/73 (4.1)
Obstetric APS, (20)	4/20 (20.0)	0/20 (0)
VITT, (3)	3/3 (100)	3/3 (100)
COVID-19 with thrombosis, (10)	0/10 (0)	0/10 (0)
COVID-19, without thrombosis, (10)	0/10 (0)	0/10 (0)
SLE, (20)	0/10 (0)	0/10 (0)
Non-thrombotic APS, (20)	0/10 (0)	0/10 (0)
Healthy donors, (45)	0/45 (0)	0/45 (0)

APS, antiphospholipid syndrome; SLE, systemic lupus; VITT, vaccine-induced immune thrombotic thrombocytopenia.

### Clinical associations of anti-β2-GPI/PF4 antibodies in thrombotic APS patients

Analyzing the possible association between the presence of autoantibodies and clinical manifestations, a significant association between positivity for anti-β2-GPI/PF4 antibodies and the development of venous thrombosis (p=0.032) was found. In APS, the onset of deep venous thrombosis appeared to be more frequent in patients who were positive for anti-β2-GPI/PF4 antibodies compared to those who were negative (p = 0.049). No significant differences emerged for the development of arterial thrombosis events and extra-criteria manifestations (**Table 3**).

**Table 3:** Anti-β2-GPI/PF4 antibodies prevalence according to the demographic and clinical manifestations.

Characteristics	Thrombotic APS patients		*P-value*
	*Anti-β2-GPI/PF4 antibodies +*	*Anti-β2-GPI/PF4 antibodies –*	
female, n (%)	17 (68%)	34 (70.8%)	*0.80*
male, n (%)	8 (32%)	14 (29.2%)	*0.80*
Median age in years (IQR)	47 (38-51)	58 (48-67)	
Median age of onset (IQR)	31 (28-43)	44.5 (36.5-51)	
PAPS, n (%)	15 (60%)	29 (60%)	*0.97*
SAPS, n (%)	9 (36%)	18 (37.5%)	*0.89*
Catastrophic syndrome, n (%)	1 (4%)	1 (2.5%)	*0.63*
Arterial Thrombosis	13 (52%)	36 (75%)	*0.23*
Venous Thrombosis	19 (76%)	28 (58.33%)	***0.032***
Recurrent Thrombosis, n (%)	14 (56%)	20 (41.66%)	*0.24*
Extra-criteria manifestations, n (%)	15 (60%)	23 (47.91%)	*0.97*

The bold value indicates the number of patients.

**Figure 3:**
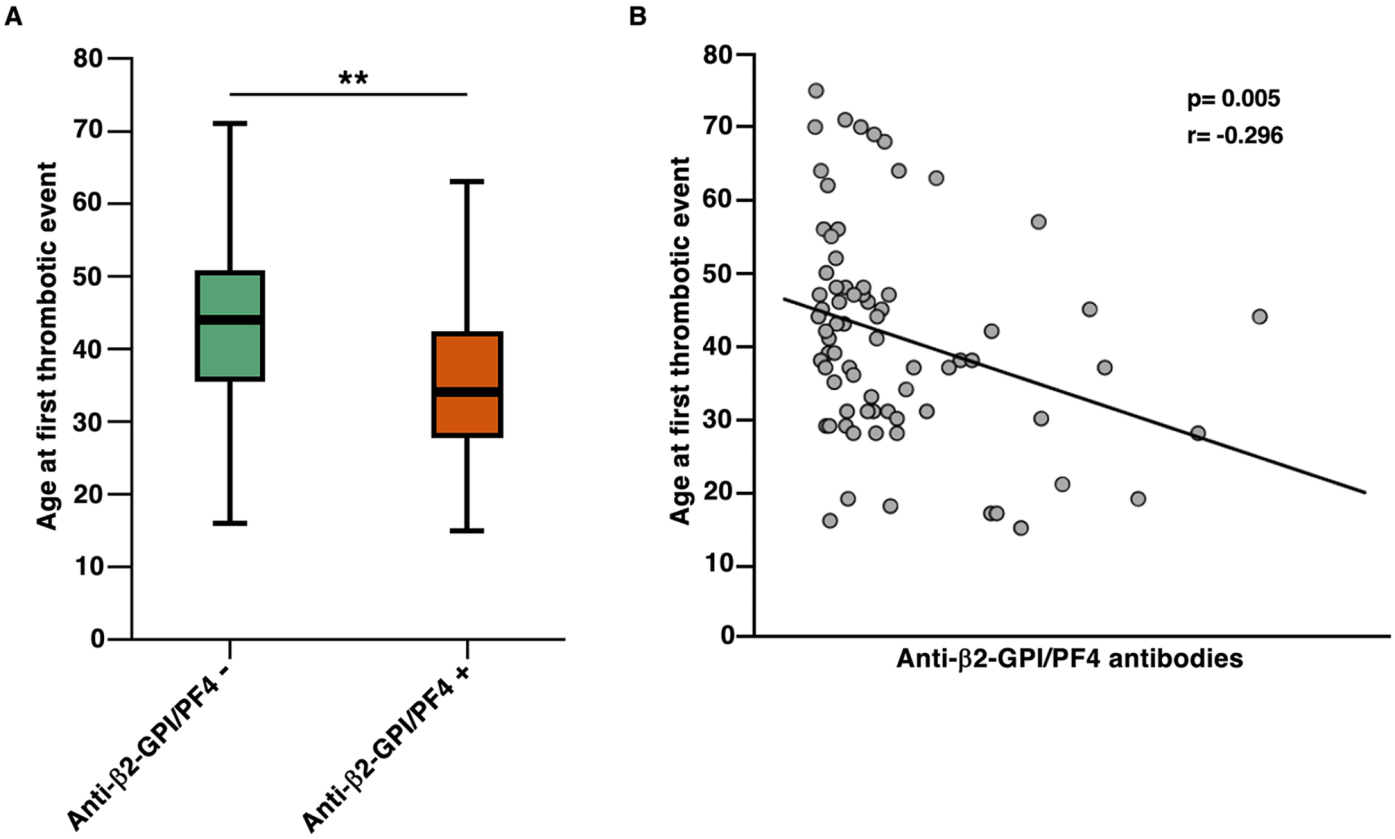
Correlation between thrombotic manifestations and anti-β2GPI/PF4 antibodies. **(A)** Significant positivity of anti-β2GPI/PF4 antibodies in patients who experienced the first thrombotic event at a younger age. **p < 0.01. **(B)** A linear negative correlation between anti-β2GPI/PF4 antibodies and the age of the first thrombotic event.

Data analysis of anti-β2-GPI/PF4 antibody prevalence according to demographic and clinical manifestations also revealed a significant association between the onset of APS before the age of 35 and anti-β2-GPI/PF4 antibody positivity (p = 0.01) (**Figure 3**). In addition, the antibody titer was higher in patients with a younger age (p < 0.001). On the contrary, no significant gender difference was found. Finally, the anti-β2-GPI/PF4 antibody titer correlated with aβ2-GPI and aCL antibodies (p < 0.001). An association was found between positivity for anti-2GPI/PF4 antibodies and triple positivity to conventional aPLs (p = 0.028), primarily because the majority of patients were negative for both.

### IgG fractions from thrombotic APS patients induce signalling activation pathways and TF expression in platelets

**Figure 4:**
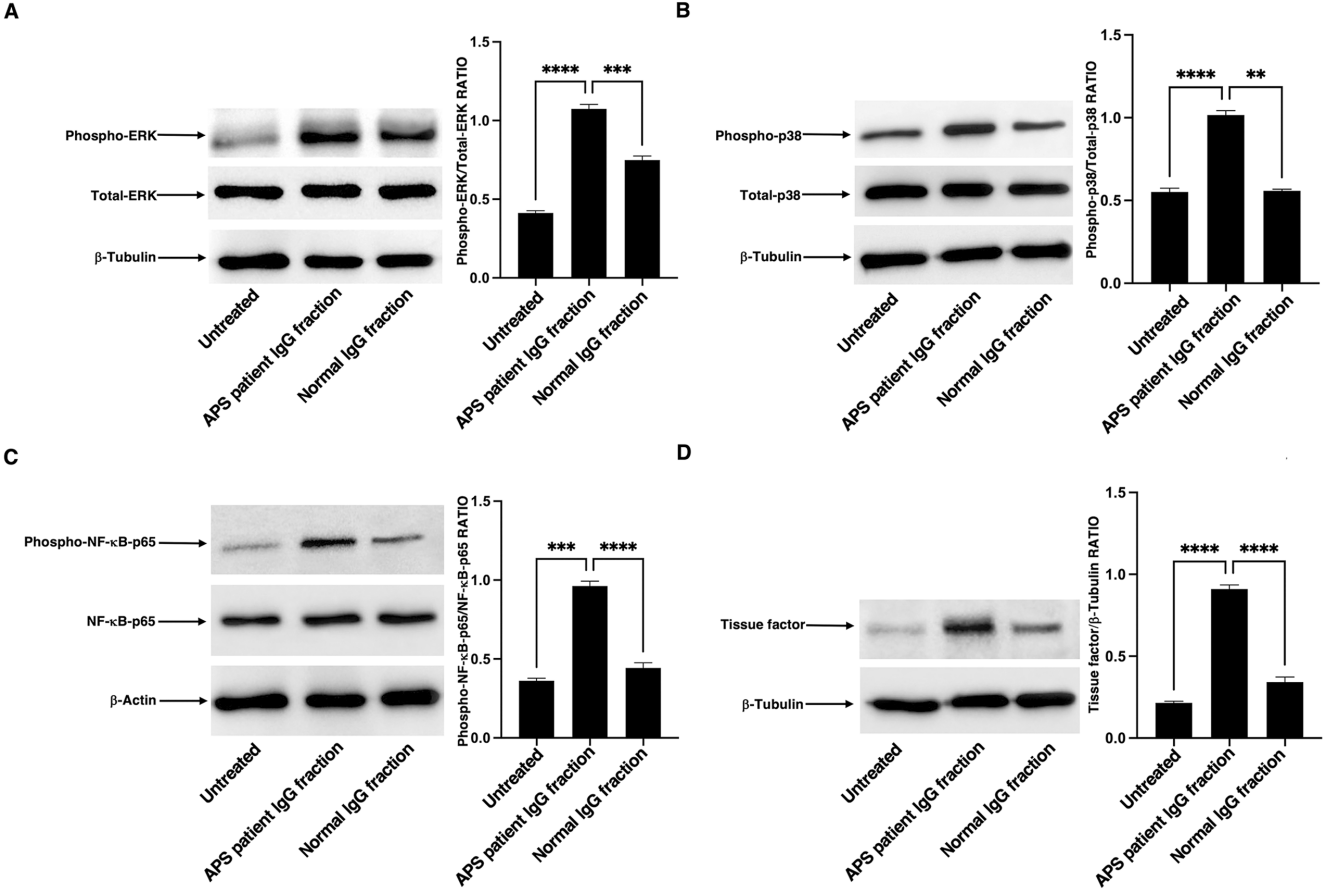
Analysis of MAPK signalling activation and TF expression in platelets stimulated with IgG fractions specific for β2-GPI/PF4 complex from thrombotic APS patients. Human platelets from HDs untreated or stimulated with IgG fractions specific for β2-GPI/PF4 complex from thrombotic APS patients were lysed and analysed by western blot. **(A)** Phospho-ERK expression using rabbit anti-phospho-ERK1/2 antibody. **(B)** Phospho-p38 expression using rabbit anti-phospho-p38 antibody. **(C)** phospho-NF-κB-p65 expression using rabbit anti-phospho-NF-κB-p65 antibody. **(D)** TF expression using rabbit anti-TF antibody. Anti-β-tubulin or anti-β-actin antibodies were used to evaluate loading controls. Densitometric phospho-proteins/total proteins ratios **(A–C)** and TF/β-tubulin ratios are shown in the right panel. Data are reported as mean (S.D.) from three independent experiments. Statistical analysis indicated: **p < 0.01; ***p < 0.001; ****p < 0.0001.

To evaluate a possible functional effect of anti-β2-GPI/PF4 antibodies, we investigated the activation of signalling pathways in platelets, whose role is known in immune-mediated thrombotic manifestations. Therefore, platelets from HDs were treated with IgG fractions (specific for β2-GPI/PF4 complex) isolated from patients with thrombotic APS and, as a negative control, with IgG fractions from normal human serum. As shown in **Figure 4**, treatment of platelets with the IgG fractions from thrombotic APS induced a significant increase of phospho-ERK (**Figure 4A**), phospho-p38 (**Figure 4B**) and phospho-NF-κB-p65 (**Figure 4C**) expression, as compared to control untreated platelets or platelets treated with normal IgG fractions. In parallel experiments, as a consequence of the activation of these molecules, a significant increase of TF expression (**Figure 4D**), following thrombotic APS IgG fraction stimulation, has also been demonstrated. Conversely, virtually no reactivity was observed in untreated cells or in cells stimulated with normal IgG fractions.

## Discussion

In this study, the presence of anti-β2-GPI/PF4 antibodies has been identified in a group of APS patients. Our findings add novel insights to the immunopathogenic role of these antibodies in APS, particularly their potential contribution to platelet activation.

Proulle et al. demonstrated that platelets are essential for the prothrombotic effects of anti-β2-GPI autoantibodies in a murine model of APS (33). Indeed, cell binding of fluorescently labelled anti-β2-GPI autoantibodies to β2-GPI demonstrated their colocalization on the platelet thrombus, but not the endothelium. Again, anti-β2GPI autoantibodies amplified platelet activation, monitored by calcium mobilization and endothelial activation, identified by intercellular adhesion molecule-1 expression (ICAM-1). The aβ2-GPI/β2-GPI autoantibody complex binds to the thrombus, enhancing platelet activation signalling pathways as a consequence of interaction with phospholipids on the cell surface (phosphatidylserine, phosphatidylcholine) or with receptors of the platelet membrane (34, 35). In our previous paper we demonstrated that aPLs, in particular anti-β2-GPI antibodies, were able to trigger intracellular signals in platelets, involving IRAK phosphorylation and NF-kB activation, inducing the up-regulation of TF, the major initiator of the clotting cascade (36, 37). Therefore, platelets are the primary target of the complexes, such as the aβ2-GPI/β2-GPI autoantibody complex or anti-β2-GPI/PF4 complex, and products released by activated platelets are responsible for activating local endothelial cells. In fact, inhibition of platelet activation prevents the activation of endothelial cells (38, 39).

The main significance of the interaction between the two proteins is the stabilization of β2-GPI dimers following oxidation, attributed to PF4 binding (16). This may facilitate antibody recognition of novel and/or cryptic epitopes of β2-GPI. Nevertheless, one limitation of our study is that the partial reduction in reactivity could reflect a non-specific interaction between PF4 and antibodies that recognize oxidized B2-GPI.

In our study levels of anti-β2-GPI/PF4 antibodies were significantly higher in APS patients than in HDs, suggesting a possible pathogenic role for the antibodies in the syndrome, where the immune-mediated activation of platelets may contribute to the prothrombotic state. Upon analyzing the data, a positive linear correlation emerged between the presence of the antibodies not only with disease onset at a younger age compared to epidemiological data in the literature, but also with the titer of anti-β2-GPI/PF4 appearing to be higher in patients at a younger age both at diagnosis and at the current evaluation. Interestingly, we also detected the presence of anti-β2-GPI/PF4 antibodies in all 3 patients with VITT, but not in patients with COVID-19, including those with thrombophilic complications, suggesting that the thrombotic mechanism underlying VITT is different from that of COVID-19 infection. This finding rekindles interest in the association between APS and VITT, emphasizing the role of platelet activation as a fundamental element of thrombophilia in both conditions. Indeed, several authors suggested that platelets play a central role in the pathogenesis of both APS and VITT (25, 34–36), since platelet secretion leads to increased endothelial activation and fibrin generation (33). Interestingly, in VITT patients, the signs and symptoms correlate with the high level of antibodies to anti-PF4-cationic complex antibodies capable of activating platelets obtained from healthy donors (28, 29, 39–43). Furthermore, in a previous study we observed that IgG fractions from VITT patients were capable of triggering a signaling pathway involving the activation of ERK and p38 MAPK, which can lead to increased TF expression, resulting in amplification of platelet activation and the coagulation cascade (44).

It could be intriguing to explore the mechanism by which anti-β2-GPI/PF4 antibodies are generated and may induce platelet activation. In this concern, we demonstrated that the antibodies obtained from APS patients induced platelet activation, as revealed by a significant increase of phospho-ERK and phospho-p38 expression, NF-κB activation, with consequent TF expression. These functional data can be interpreted in the context of polyclonality for APS; it may represent a limitation, although the observed activation pattern (ERK, p38, NF-κB, TF) is in line with mechanisms previously described both for IgG fraction and for antibodies purified from serum of APS patients (36–38). It is reasonable to assume that these specific mechanisms, driven by anti-β2-GPI/PF4, converge to create a pro-thrombotic state, which can manifest with arterial thrombosis.

However, the main limitation of this study was its monocentric design. Given the data obtained from our work, further studies involving a larger and multicentric cohort of patients would be necessary to validate the role of anti-β2-GPI/PF4 antibodies in diagnosing patients with APS, clinical associations, and risk stratification. It would be interesting to explore the antibody’s role in outlining the existence of an immunological profile in patients with early-onset disease.

From a translational perspective, the detection of anti-β2-GPI/PF4 antibodies could enrich the current serological criteria of APS, especially in patients with atypical or refractory thrombotic profiles.

## Data Availability

The raw data supporting the conclusions of this article will be made available by the authors, without undue reservation.
