# Supplementary material for: Antigenic assessment for the β2-glycoprotein I/Platelet factor 4 complex in thrombotic patients with antiphospholipid syndrome
Source: Front Immunol. 2026 Jan 12;16:1674181. doi: 10.3389/fimmu.2025.1674181 (PMC12832691; doi:10.3389/fimmu.2025.1674181)
Supplement: Supplementary file 1 [file DataSheet1.pdf]

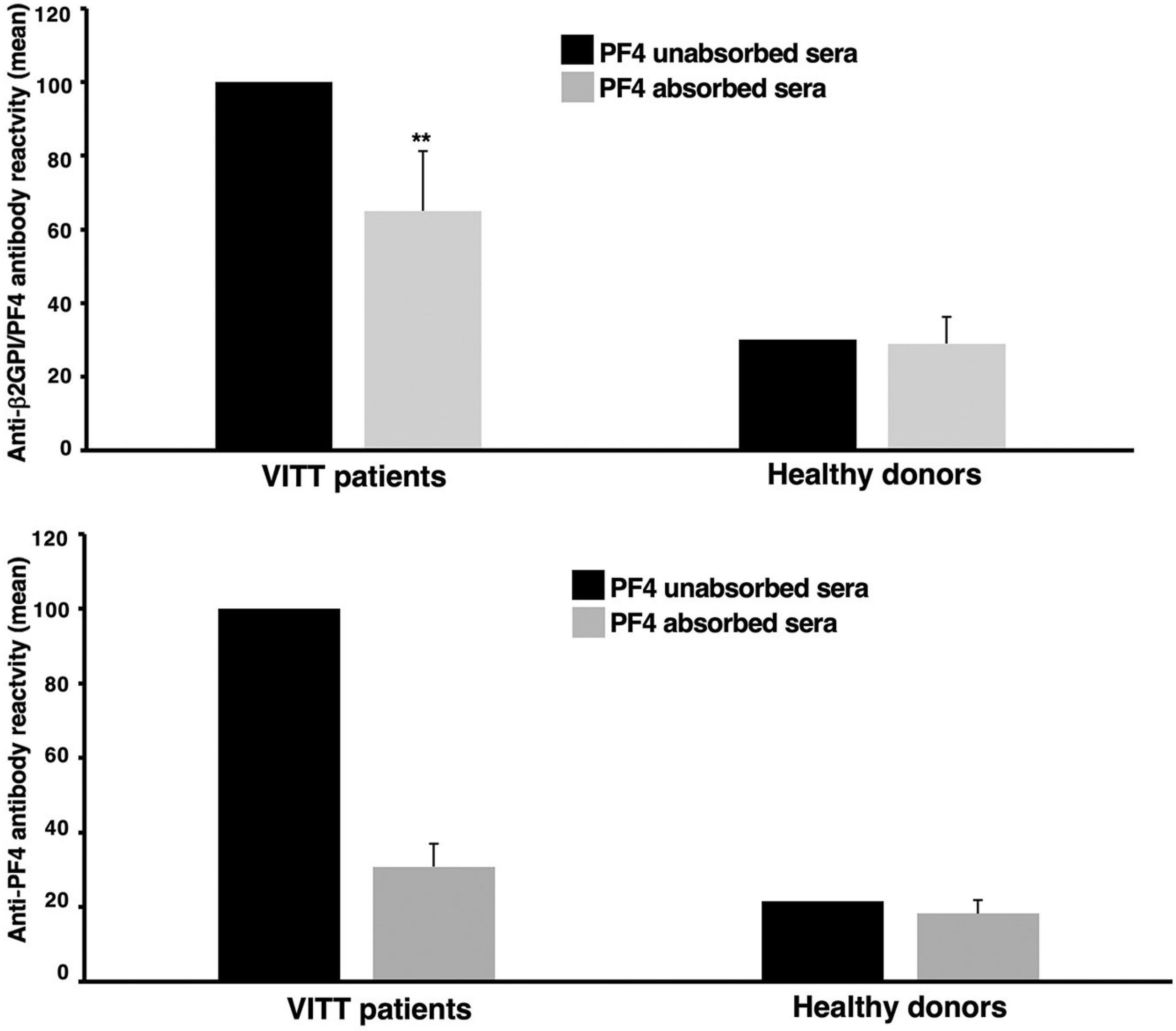

Figure S1. **Absorption tests for detection of anti-β2-GPI/PF4 antibody specificity.** Sera of patients with VITT (positive for both anti-β2-GPI/PF4 and anti-PF4 antibodies) and of healthy donors, unabsorbed or absorbed with PF4, were analyzed by ELISA for the detection of anti-β2-GPI/PF4 antibodies (upper bar graph) and for anti-PF4 antibodies (lower bar graph). Absorbances of unabsorbed patients' sera were set to 100%. ** $p = 0.01$ *versus* reactivity with PF4 of absorbed patients' sera. Therefore, no significant inhibition of reactivity was observed after PF4 absorption, (upper bar graph). On the contrary, as expected, the reactivity to PF4 of absorbed sera was significantly reduced. These data demonstrate a specific recognition by anti-β2-GPI/PF4 antibodies detected in VITT patient sera.
