# Supplementary material for: Antigenic assessment for the β2-glycoprotein I/Platelet factor 4 complex in thrombotic patients with antiphospholipid syndrome
Source: Front Immunol. 2026 Jan 12;16:1674181. doi: 10.3389/fimmu.2025.1674181 (PMC12832691; doi:10.3389/fimmu.2025.1674181)
Supplement: Supplementary file 2 [file DataSheet2.pdf]

LAC	aCL IgG	aCL IgM	aβ2GPI IgG	aβ2GPI IgM	aPF4-β2GPI
2	2	2	2	2	2
2	2	2	2	2	2
0	1	0	0	0	0
2	2	2	2	2	2
2	2	2	2	2	2
2	2	1	2	1	2
2	2	1	2	1	2
2	2	1	2	0	2
2	2	1	2	0	2
2	2	1	2	0	2
2	2	1	2	0	2
2	2	1	1	2	2
2	2	1	1	1	0
2	2	1	1	0	2
2	2	0	2	0	0
2	2	0	2	0	0
2	2	0	0	0	2
2	1	1	1	1	2
2	1	1	1	1	0
2	1	1	0	1	2
2	1	1	0	0	2
2	1	1	0	0	0
2	1	1	0	0	0
2	1	0	2	0	0
2	1	0	0	0	0
0	1	0	0	0	0
2	0	0	1	1	0
2	0	0	1	0	0
0	0	0	1	1	0
0	1	0	0	0	0
2	0	0	0	0	2
2	0	0	0	0	0
2	0	0	0	0	0
2	0	0	0	0	0
2	0	0	0	0	0
2	0	0	0	0	0
2	0	0	0	0	0
0	2	2	2	2	2
0	2	2	2	2	2
0	2	2	2	1	0
0	2	0	2	0	0
0	2	0	2	0	0
0	2	0	2	0	0
0	2	0	0	0	0
0	2	0	0	0	0
0	2	0	0	0	0
0	2	0	0	0	0
0	1	2	2	1	0
0	1	2	2	1	0
0	1	1	2	0	2
0	1	1	1	1	2
0	1	1	1	1	2
0	1	1	1	1	0
0	1	1	1	0	0
0	1	1	0	1	0
0	1	1	0	1	0
0	1	1	0	0	0
0	1	1	0	0	0
0	1	0	2	0	2
0	0	0	0	0	0
0	0	0	0	0	0
0	1	0	2	0	0
0	1	0	2	0	0
0	1	0	1	2	2
0	1	0	1	1	0
0	1	0	1	0	2
0	1	0	1	0	0
0	1	0	1	0	0
0	0	0	0	1	0
0	0	0	1	1	0
0	0	0	0	0	0
0	0	0	0	0	0
0	0	0	0	0	0

Figure S2. Heatmap showing all thrombotic APS patients results.

Obtained results were separated by tested antibody (horizontal). The legend (colors) represents the titers of each aPL (green negative result; orange represents low levels, red represents high levels). The results of the same patients are shown in the horizontal line. Therefore, aPL and anti-β2-GPI/PF4 test can be compared directly.
