# Supplementary material for: Antigenic assessment for the β2-glycoprotein I/Platelet factor 4 complex in thrombotic patients with antiphospholipid syndrome
Source: Front Immunol. 2026 Jan 12;16:1674181. doi: 10.3389/fimmu.2025.1674181 (PMC12832691; doi:10.3389/fimmu.2025.1674181)
Supplement: Supplementary file 3 [file Table1.docx]

**Supplementary Table 1. Demographics and clinical characteristics of 3 vaccine-induced immune thrombotic thrombocytopenia (VITT) post-administration of ChAdOx1 nCoV-19 vaccine.**

|  | **Patient 1** | **Patient 2** | **Patient 3** |
| --- | --- | --- | --- |
| **Gender** | Female | Female | Male |
| **Age** | 57-year-old | 55-year-old | 59-year-old |
| **Preexisting conditions** | Hypothyroidism, in follow-up care after breast cancer treated surgically in 2012 | Hypothyroidism | BMI > 30  Hypertension |
| **Vaccine** | ChAdOx1 nCoV-19 | ChAdOx1 nCoV-19 | ChAdOx1 nCoV-19 |
| **ΔT Vaccine** | 9 days after the I dose | 7 days after the I dose | 5 days after the I dose |
| **Clinical manifestations** | left hemiplegia, right gaze deviation, dysarthria, and left neglect | abdominal pain, transient episode of aphasia and right hemiparesis, followed 2 h later by generalised seizures and coma | thoraco-abdominal pain and epigastric pain |
| **Angio-CT scan** | right middle cerebral artery occlusion | occlusion of the right internal carotid artery terminus and of the left MCA, extensive ischemic cores and severe bilateral hypoperfusion, without treatable penumbra | total Thrombosis of the intra extra hepatic portal system and partial Thrombosis of the superior mesenteric and collaterals |
